# Inter-Individual Differences Explain More Variance in Conditioned Pain Modulation Than Age, Sex and Conditioning Stimulus Intensity Combined

**DOI:** 10.3390/brainsci11091186

**Published:** 2021-09-09

**Authors:** Philipp Graeff, Alina Itter, Katharina Wach, Ruth Ruscheweyh

**Affiliations:** 1 Graduate School of Systemic Neuroscience, Ludwig-Maximilians-University Munich, 82152 Planegg, Germany; ruth.ruscheweyh@med.uni-muenchen.de; 2 Research Training Group (RTG) 2175 Perception in Context and Its Neural Basis, Ludwig-Maximilians-University Munich, 82152 Planegg, Germany; 3 Department of Neurology, University Hospital Großhadern, Ludwig-Maximilians-University Munich, 81377 Munich, Germany; a.itter@campus.lmu.de (A.I.); katharina.wach@gmx.de (K.W.)

**Keywords:** conditioned pain modulation, endogenous analgesia, conditioning stimulus, interindividual factors, CPM variability

## Abstract

Conditioned pain modulation (CPM) describes the reduction in pain evoked by a test stimulus (TS) when presented together with a heterotopic painful conditioning stimulus (CS). CPM has been proposed to reflect inter-individual differences in endogenous pain modulation, which may predict susceptibility for acute and chronic pain. Here, we aimed to estimate the relative variance in CPM explained by inter-individual differences compared to age, sex, and CS physical and pain intensity. We constructed linear and mixed effect models on pooled data from 171 participants of several studies, of which 97 had repeated measures. Cross-sectional analyses showed no significant effect of age, sex or CS intensity. Repeated measures analyses revealed a significant effect of CS physical intensity (*p* = 0.002) but not CS pain intensity (*p* = 0.159). Variance decomposition showed that inter-individual differences accounted for 24% to 34% of the variance in CPM while age, sex, and CS intensity together explained <3% to 12%. In conclusion, the variance in CPM explained by inter-individual differences largely exceeds that of commonly considered factors such as age, sex and CS intensity. This may explain why predictive capability of these factors has had conflicting results and suggests that future models investigating them should account for inter-individual differences.

## 1. Introduction

Conditioned pain modulation (CPM) paradigms measure the component of human endogenous pain inhibition underlying the “pain inhibits pain” phenomenon [1], based on a noxious test stimulus (TS) being perceived as less painful if presented in combination with a painful heterotopic conditioning stimulus (CS). CPM magnitude is reduced in a variety of chronic pain conditions, pointing towards dysregulation of endogenous pain inhibition in these patients [2].

Individual differences in CPM are considerable, and have been proposed to predict susceptibility to acute and chronic pain [3,4]. Some individual factors influencing CPM magnitude have been identified: e.g., some studies have found a larger CPM effect in males than females [5,6] and in younger compared to older subjects [7,8]. An effect of pre-existing psychological factors has been discussed, but a recent study has not shown a clear relation to the CPM effect [9]. It is currently not known how much individual variance remains after accounting for the effects of age and sex.

In addition, many different experimental paradigms have been used [10] which may also influence CPM magnitude., e.g., the role of conditioning stimulus intensity has been investigated repeatedly with inconsistent results. While some studies find no effect [11,12,13], others find larger CPM with stronger conditioning stimuli [14,15,16]. It has been proposed that as long as it is clearly painful, further increases in conditioning stimulus intensity do not increase CPM magnitude [12,13]. It might also occur that conditioning stimulus intensity does have an effect in within-subject designs [14,17], but that the effect is small compared to inter-individual differences, which makes it difficult to detect in cross-sectional designs. In addition, it is worth considering whether physical stimulus intensity or rather subjective pain perception of the conditioning stimulus is related to CPM magnitude.

It would therefore be useful to estimate the relative importance of the various factors influencing the CPM effect. There are now methods to estimate variance contributions of both fixed effects (such as sex, age and conditioning stimulus intensity) and random effects (such as remaining individual differences) within the same model [18,19], in addition to the relative variance contributions of the different fixed effects [20,21].

Here, we used pooled datasets from various studies measuring the CPM effect in healthy individuals once or multiple times to assess the relationship between CPM effect and age, sex, and conditioning stimulus physical or pain intensity in both cross-sectional and repeated measures settings, and estimated the relative variance in CPM magnitude explained by remaining inter-individual differences vs. age, sex, and conditioning stimulus intensity.

## 2. Materials and Methods

### 2.1. Pooled Data

Data was pooled from seven separate studies performed by our group, which investigated different aspects of endogenous pain inhibition in healthy participants, including at least one measurement of the conditioning pain modulation effect. Four studies included repeated measures gathered on different days. In total, data was pooled from 171 participants for cross-sectional analysis and from 97 participants for repeated-measures analysis. Of the repeated measures data pool, 83 participants had two repeated measures and 14 participants had three repeated measures.

Pooled data included three types of test stimulus: a 30 s or 60 s heat stimulus or electrical stimulation of the sural nerve. Conditioning stimulus in all studies was a cold pressor test of varying length (60 s, 90 s, 120 s). An overview of the studies can be found in Table 1.

### 2.2. Participants

All studies were conducted in accordance with the Declaration of Helsinki and were approved by the ethics committee of the Ludwig-Maximilian University, Munich. Healthy participants were recruited by announcements on the university campus and gave written informed consent. Participants had to meet the following criteria (which apply to all our studies with healthy participants): (1) age ≥18 years, (2) sufficient knowledge of German, (3) no severe internal, neurological or psychiatric conditions, (4) no history of chronic pain, (5) no alcohol, nicotine or drug abuse, (6) no regular medication (except hormonal contraception or thyroid hormones), (7) not pregnant or breastfeeding, (8) no acute pain and no use of pain medication within the previous 48 h, (9) Beck’s depression inventory score <13.

### 2.3. Conditioned Pain Modulation

The study data collected utilized three different CPM paradigms (combinations of test stimulus and conditioning stimulus).

The conditioning stimulus in all studies was immersion of the contralateral (in regard to the test stimulus) hand into a Styrofoam box filled with cold water for 60–120 s. Pain intensity ratings were collected on an 11-point numeric rating scale (NRS, 0 = no pain, 10 = most intense pain imaginable). According to the results of Granot [12], we aimed at a conditioning stimulus intensity that was clearly painful (usually ≥3 on the NRS) but could be tolerated for the planned stimulus duration. To achieve this, in a pre-test, water temperature was individually adjusted starting at 10 °C.

Test stimuli were either painful heat (30 s or 60 s) or electrical stimulation of the sural nerve.

Heat stimulation was applied via a thermode (Pathway system, Medoc, Israel) to the volar side of the forearm at a temperature individually tailored to evoke a pain intensity rating of approximately 6 on the NRS, resulting in temperatures of 46.3 ± 1.2 °C, range: 43–49 °C. Heat pain intensity ratings were collected every 10 s for the stimulus duration. The heat stimulus was first presented in isolation (baseline) and then 30 s following the start of the conditioning stimulus. A ≥ 5 min break was taken between baseline and conditioning measures and the thermode was shifted between measurements to avoid habituation.

Painful electrical stimulation of the sural nerve was performed as described previously [23,24]. Electrical stimuli were applied every 8–12 s for three consecutive 2 min blocks, each block containing 12 stimuli. Conditioning stimulus was present during the second block (120 s). Pain intensity rating of the test stimulus was collected at the end of each block as the average pain intensity of the last five stimuli.

CPM effect was calculated as the percentage difference between average test stimulus NRS rating at baseline (NRSts(baseline)) and during conditioning stimulation (NRSts(cond)), where a more negative result denotes a stronger CPM effect:

$$\mathrm{CPM}\;\mathrm{effect}=\frac{\mathrm{NRSts}(\mathrm{cond})-\mathrm{NRSts}\left(\mathrm{baseline}\right)}{\mathrm{NRSts}\left(\mathrm{baseline}\right)}$$

### 2.4. Statistical Analysis

All statistical analysis was performed in R [25]. *p* < 0.05 (two-sided) was considered statistically significant.

Linear regression: linear regression analyses were performed using the *lm()* function of the *stats* package [25]. The linear regression models used for the cross-sectional population analysis of the relationship between CPM effect and age, sex, paradigm and conditioning stimulus pain or physical intensity were:

CPM effect ~ NRS_cond_ + age + sex + paradigm (1)

CPM effect ~ temperature_cond_ + age + sex + paradigm (2)

NRS_cond_ describes the CS pain intensity on the NRS (0–10) immediately after the test stimulus, temperature_cond_ describes the physical intensity (cold water temperature) of the CS and paradigm describes the CPM paradigm as a factor. The three paradigms included were 60 s heat/90 s cold (reference), 30 s heat/60 s cold, and Electrical/120 s cold.

Mixed models: linear mixed model analysis was performed on the pooled repeated measures data using the lme4 [26] and car [27] packages in R. From the repeated measures population, we extracted those participants in which the CS pain intensity differed by at least 0.5 points on the NRS between measurements (85 participants, 184 observations) and those participants in which the CS physical intensity (temperature) differed by at least 0.5 °C between measurements (52 participants, 118 observations). Linear mixed models were constructed for the pain intensity rating variable, and the temperature variable subgroup using the *lmer()* function of lme4 [26]:

CPM effect ~ NRS_cond_ + age + sex + paradigm + repeat + (1|participant) (3)

CPM effect ~ temperature_cond_+ age + sex + paradigm + repeat + (1|participant) (4)

CPM effect, NRS_cond_ and temperature_cond_ are as described above; paradigm describes the two CPM paradigms included in the dataset: 30 s heat/60 s cold and Electrical/120s cold (reference). Repeat describes the measurement repeat (i.e., first, second, or third measurement for that participant). The random effect (1|participant) allows for variable intercept for each participant. Significance of each fixed effect was tested using Wald’s Chi-squared test implemented via the *Anova()* function of the *car* package [27].

Fixed vs. random effects variance contribution: in order to determine the inter-individual variability of the CPM effect not explained by age and sex we calculated the percent variance in CPM effect explained as contributed by fixed and random effects. To do this we calculated the marginal and conditional coefficient of determination as described in Nakagawa et al. [18,19] using the *r.squaredGLMM()* function of the *MuMIn* package [28] on the mixed models described above. Marginal R^2^ describes the variance explained by all fixed effects and conditional R^2^ describes the variance explained by both fixed and random effects combined. Variance explained by inter-individual variability was calculated by subtracting the marginal from the conditional R^2^.

Variance contribution of the fixed effects: in order to determine the relative contribution of each fixed effect factor to the variance in the CPM effect, the *calc.relimp()* function of the *relaimpo* package was used [29]. We utilized the “lmg” option of R^2^ variance decomposition, which averages the R^2^ contribution of each factor over all orderings as described by Lindeman, Merenda and Gold [21] and Chevan and Sutherland [20].

As *calc.relimp()* cannot handle mixed model input, we constructed the following linear models using the *lm()* function to reflect the fixed effects of the mixed model 3 and 4 and used them as input to the *calc.relimp()* function:

CPM effect ~ NRS_cond_ + age + sex + paradigm + repeat (5)

CPM effect ~ temperature_cond_ + age + sex + paradigm + repeat (6)

## 3. Results

### 3.1. Cross-Sectional Analysis

The mean age of the cross-sectional sample was 29 ± 11 (n = 171, 64 women). The average CPM effect was significant (*p* < 0.001) and amounted to -16.9 ± 23.9%. The average pain rating of the conditioning stimulus was 4.5 ± 1.8 (range: 1.0–9.0) on the NRS and the average temperature of the conditioning stimulus was 7.9 ± 3.4 °C (range: 0.1–16.2).

**Figure 1:**
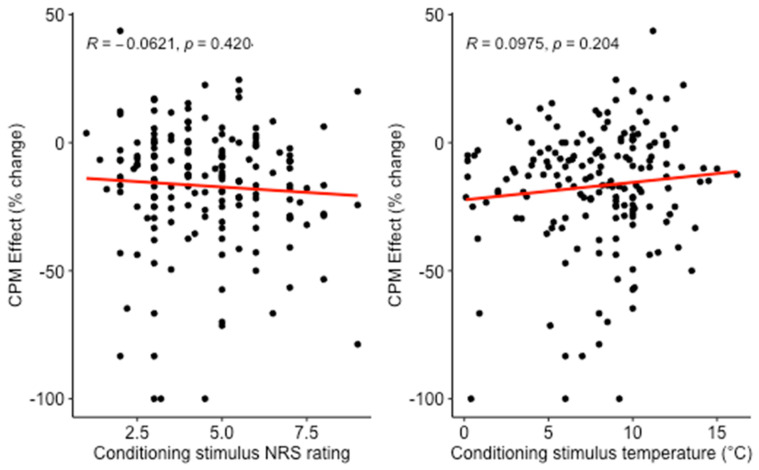
Relation between CPM effect and conditioning stimulus pain intensity or conditioning stimulus temperature in the cross-sectional analysis. Conditioning stimulus was hand immersion in cold water. More negative CPM effect designates a larger reduction of test pain rating by the conditioning stimulus.

Multiple linear regression was calculated to predict CPM effect from participant age, sex, CPM protocol and either CS pain or physical intensity (models 1 and 2, Table 2). There was no significant relation of the CPM effect with CS pain intensity (NRS_cond_) or CS physical intensity (temperature_cond_) (Figure 1). There also was no significant relation of age, sex or CPM paradigm with the CPM effect. Proportions of variance in CPM effect explained by all predictors together were low (1.1% for model 1 and 1.9% for model 2). Results for the individual predictors are given in Table 2.

### 3.2. Repeated Measures Analysis of Linear Mixed Models

The average CPM effect in the repeated measures sample (Model 3: 184 observations, Model 4: 118 observations) was significant (both *p* < 0.001) and amounted to −17.6 ± 24.6% and −16.9 ± 21.2%, respectively. Mean difference in NRS rating of CS between observations in Model 3 was 1.9 ± 1.2. Mean difference in CS temperature between observations in Model 4 was 4.3 ± 2.6 °C.

Linear mixed models were constructed in order to analyze the contributions of the different fixed effects to the CPM effect (Table 3). A larger (i.e., more negative) CPM effect was significantly related to a lower CS temperature (*p* = 0.001, −1.5% change in CPM effect per °C temperature decrease) in Model 4.

The remaining relations were all non-significant (Table 3): in Model 3, CPM effect increased (i.e., was more negative) by −1.5% per NRS point with increasing CS pain intensity (*p* = 0.159). CPM effect decreased non significantly by 0.26% per year of age in both models. CPM effect in women was 2.5% lower in Model 3, and 1.9% larger in Model 4 compared to men (both n.s.). The 30 s heat/60 s cold protocol produced a 7.8% (Model 3) and 8.5% (Model 4) larger CPM effect than the electrical/120 s cold protocol (both n.s.).

### 3.3. Repeated Measures Analysis: Decomposition of Explained Variance

To determine the relative variance explained by fixed effects vs. inter-individual differences we decomposed the total R^2^ into conditional (fixed effects) and marginal (fixed effects + inter-individual factors) R^2^. Conditional R^2^ and marginal R^2^ were 3.4% and 27.4% in Model 3, and 11.5% and 45.8% in Model 4, respectively. Therefore, in Model 3, all fixed effects together explained 3.4% of the variance in the CPM effect, while the remaining inter-individual differences explained 24.0%. In Model 4, all fixed effects together explained 11.5% of the variance in the CPM effect and remaining inter-individual differences explained 34.3%.

**Figure 2:**
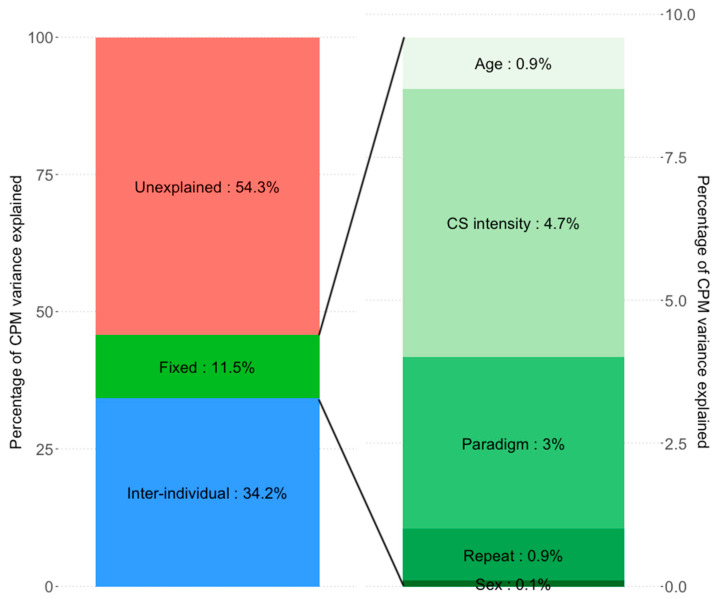
Proportions of variance in CPM explained by the CS physical intensity models (models 4 and 6)**.** Inter-individual differences explain substantially more CPM variance than the fixed effects of age, sex, CS intensity or the nuisance regressors CPM paradigm or measurement repeat (34.2% vs. 11.5%, respectively). Of the fixed effects, CS intensity explains the most variance (4.65%), followed by CPM paradigm (3.01%). Age (0.89%), Sex (0.09%), and measurement repeat (0.87%) explain negligible amounts. Due to different model types (model 4: linear mixed effects model; Model 6: multiple linear regression model) and different statistical methods needed to estimate partial variance explained, the sum of fixed effects variance explained in Model 6 does not equal exactly the estimated variance of combined fixed effects in Model 4.

Finally, in order to further decompose the variance explained by the different fixed effects, we constructed linear models including only the fixed effects (Models 5 and 6, Table 4). CS pain intensity explained 0.7% of the variance (model 5) while CS physical intensity (cold water temperature) explained 4.7% (Model 6). The type of CPM paradigm used explained 1.5% and 3.0% of the variance in Model 5 and 6, respectively. Age, sex and measurement repeat made only small contributions to the explained variance (<1% each). Variance breakdown of the significant model (Model 4) and the relative variances of its fixed effect are seen in Figure 2. It is important to note that the fixed effects’ variance in Models 5 and 6 does not sum to the variance explained by fixed effects in Models 3 and 4, respectively. This is due to the fact that the models used differ, resulting in slightly different fits. Additionally the statistical methods used for variance decomposition in the two types of analysis are different, which will further lead to discrepancies.

## 4. Discussion

Main results of the present study were:

(i) In a large cross-sectional analysis, neither CS physical intensity nor CS pain intensity predicted the CPM effect. In contrast, in a repeated measures analysis, CS physical intensity, but not CS pain intensity predicted the CPM effect.
(ii) Inter-individual differences explained a large proportion of CPM variance (24.0% to 34.2%) while all fixed effects together (CS pain or physical intensity, age, sex, CPM paradigm, measurement repeat) predicted only 3.4% to 11.5% of CPM variance.

### 4.1. Conditioning Stimulus Physical Intensity and Pain Intensity

Previous results on the dependence of CPM magnitude on CS intensity are inconsistent, ranging from no effect [11,12,13] to a significantly increased CPM effect with higher CS intensity [14,15,16]. Our study confirms and extends previous cross-sectional results [12] showing no significant relation between CPM magnitude and either CS physical intensity or CS pain rating in a large cross-sectional sample (n = 171). In contrast, the repeated measures analysis revealed a significant relation between CPM magnitude and CS physical intensity, while the relation with CS pain intensity remained non-significant. This raises two interesting points.

First, as CPM is a psychophysical measure, one could assume that the subjective pain experience would have a larger influence on the CPM effect than the CS physical intensity. Indeed, some studies have shown a relation between CS pain intensity and CPM magnitude [15,16]. In addition, placebo-induced reduction of perceived CS pain was related to a reduced CPM effect [30] and CS-induced supraspinal activation correlated with CPM magnitude [31]. On the other hand, some processes underlying CPM seem to be independent of the subjective pain experience [32]. In spite of the above cited supraspinal influences, a spino-bulbo-spinal pathway is thought to be the main circuitry responsible for CPM [33,34]. This may be one possible explanation for CS physical intensity being a larger determinant than CS pain intensity. Consistently, some previous studies have shown a relation between CS physical intensity and CPM effect [14,35]. However, since CS physical and pain intensity are highly correlated, only studies that investigate both parameters over a range of different values will be able to show which correlation is larger. The present study conducted such a direct comparison and found a preferential relation with CS physical intensity. Notably, it may be both a strength and a limitation of the present study that variability in CS physical and/or pain intensity was mostly random and not due to a dedicated study design. This point would clearly merit further investigation, systematically and independently varying both CS physical and pain intensity, ideally over more than two to three observations per subject.

Second, the significant relation between CPM magnitude and CS physical intensity was detected in the repeated measures but not in the cross-sectional analysis. This suggests that within a given subject, there is a dependence of CPM magnitude on CS physical intensity, which however is small compared to inter-individual variability in the CPM effect. Analysis of explained variance indeed showed that the variance due to inter-individual differences is much larger than the variance explained by CS physical intensity (see below).

### 4.2. Age, Sex, Measurement Eepeat and CPM Paradigm

While some previous findings have suggests less efficient CPM with increasing age [7,8,36], we did not find such a relationship. This may be due to the limited age range present in our dataset, as most of our participants were young. We also found no difference in CPM effect between men and women, which is consistent with some previous findings [37,38], but larger CPM effects in men compared to women have also been reported [6,39]. We found no significant effect of the CPM paradigm used, suggesting no inherent difference between paradigms. However, we did not aim to investigate the effect of paradigm and included it as a regressor solely to control for any potential paradigm-related differences. Our data stemmed from only three different paradigms, and all used cold pain as CS. Measurement repeat was also included for control, not revealing any significant effects.

### 4.3. Variance Explained by Inter-Individual Differences vs. Fixed Variables

To our knowledge, our study is the first to estimate the relative importance of intra-individual differences (other than age or sex) vs. fixed variables for the CPM effect. By including only participant as random effect, we could estimate only the variance attributed to each individual participant, i.e., the inter-individual contribution to CPM variance. It resulted that the variance explained by inter-individual differences was large (24.0% to 34.2%) compared to that of all fixed effects combined (3.4% to 11.5%). Among the fixed effects, while the contribution of CS pain intensity, age, sex and measurement repeat were all <1%, only CS physical intensity (4.7%) and CPM paradigm (1.5% to 3.0%) made a somewhat larger contribution. This again raises two important points for discussion.

First, the large effect of inter-individual differences provides an important basis for recent attempts to use inter-individual differences as a predictor for acute or chronic pain states [3,40,41,42]. In comparison, methodological effects such as CPM paradigm and CS stimulus intensity explain much less variance. Even the contribution of commonly included factors age and sex seems to be small in comparison, with the caveat that our data were not optimally suited to detect age effects.

Second, as suggested above, the large inter-individual differences may be the reason why the relation between CS intensity and CPM effect might be difficult to detect in cross-sectional studies. Such inter-individual differences could be due to genetic, epigenetic, developmental and/or behavioral differences. Indeed, Lindstedt et al. [43] showed that genetic variation in a serotonin transporter gene is related to CPM magnitude. Cardiovascular reactivity to pain also seems to be related to CPM magnitude [44]. Psychological traits such as anxiety, depression, or catastrophizing might also contribute to inter-individual differences. Although a meta-analysis by Nahman-Averbuch et al. [9] found no link between psychological traits and overall CPM effect in healthy subjects, they did show a modality-specific relation with psychological scores of depression, anxiety and catastrophizing. Acute changes in catastrophizing and mood have been shown to influence endogenous pain inhibition [45,46]. Moreover, the role of psychological factors might be more important in clinical populations who tend to have more pronounced psychological traits. These considerations give ample room for further studies to dissect the nature of individual differences in CPM magnitude., e.g., twins studies for the role of genetic differences, and studies looking at inter-individual differences in clinical populations using a similar methodology, including psychological factors, while accounting for inter-individual differences as random factors to discern how much variance these traits account for.

Nonetheless, standardized methods are clearly desirable, and controlling for inter-individual differences in repeated measures designs may allow researchers to detect other, smaller contributing factors that would otherwise go undetected.

### 4.4. Unexplained Variance

In the present analysis, with 3.4% to 11.5% accounted for by fixed effects and 24.0% to 34.3% by inter-individual variability; this leaves 54.2% to 72.6% of the variance in CPM magnitude unexplained. Multiple factors may contribute to this. Test–retest reliability of CPM yields intraclass correlation coefficients between 0.21 and 0.82 [14,47,48] (reviewed in [49]), showing that even under constant experimental conditions, there is still a significant amount of variability between measurements. This variability may be explained in part by measurement error, which is expected when dealing with subjective pain reports. In addition, there might also be something such as the “daily form“ of the subject., e.g., transient psychological states such as acute anxiety or catastrophizing might influence CPM magnitude, especially in clinical populations. Additionally, tiredness, physical activity, menstrual phase, distraction, and previous experiences and expectations might influence the CPM effect differently between sessions. Indeed, it has been shown that factors such as distraction, catastrophizing and even voluntary mental strategies can acutely change the activity of endogenous pain inhibitory systems [24,45,50]. In addition, experimental conditions not considered in the present study, such as time of the day, gender and personality of the experimenter, or use of pain ratings compared to pain thresholds for the test stimulus might be factors that remain to be investigated.

### 4.5. Strengths and Limitations

The major strengths of our study are (1) the inclusion of a relatively large sample that allowed the comparison of cross-sectional and repeated measures effects for both CS physical and pain intensity, and (2) the use of new techniques for variance decomposition that allowed the estimation of the variance contribution of inter-individual differences and fixed effects within the same model. There are also some major limitations. First, our analysis did not include a comprehensive sample of different CPM paradigms. Therefore, it remains to be confirmed if these results translate to other CPM paradigms, especially those which assess pain thresholds instead of pain ratings for the test stimulus. Second, in our repeated measures analysis the majority of our participants only had two repeats. Repeating our analysis using multiple CS intensities and pain levels would potentially lead to more robust results. Third, our repeat analysis did not include a broad age-range, possibly precluding detection of an age effect. Lastly, our data is derived exclusively from healthy participants. It remains to be determined if our findings can be applied to clinical populations, such as chronic pain patients or patients undergoing painful medical procedures.

## 5. Conclusions

The present data emphasize the role of inter-individual differences in CPM magnitude, providing a basis for investigating these differences in clinical populations and using CPM as a predictive tool to individualize medicine by giving insight into the individuals’ endogenous pain modulation system. They also show that in comparison, CS intensity makes a minor contribution to CPM magnitude. In repeated measures designs, to further reduce methodological effects on CPM measurement, keeping CS physical intensity constant seems to be more important than CS pain intensity.

## Figures and Tables

**Table 1:** Overview of studies used for pooled data in this analysis. N = 171 participants total. Repeated measures: n = 97 participants, n = 208 observations.

Study	Age	M/F	Conditioning Stimulus	Test Stimulus	Repeated Measures	Citation
1	25 ± 6	18/12	Cold water (120 s)	Electrical	Yes	Unpublished
2	23 ± 4	15/5	Cold water (60 s)	Contact heat (30 s)	Yes	Unpublished
3	27 ± 6	14/9	Cold water (60 s)	Contact heat (30 s)	Yes	[22]
4	47 ± 10	27/0	Cold water (90 s)	Contact heat (60 s)	No	[23]
5	23 ± 5	17/9	Cold water (60 s)	Contact heat (30 s)	Yes	Unpublished
6	25 ± 5	9/19	Cold water (60 s)	Contact heat (30 s)	No	Unpublished
7	25 ± 3	7/10	Cold water (60 s)	Contact heat (30 s)	No	Unpublished

**Table 2:** Results of linear regression analysis of the cross-sectional data (n = 171). See Methods for construction of models 1 and 3. NRS_cond_^,^ CS pain intensity rating on the NRS [0–10]. Temperature_cond_, temperature of the CS (cold water bath). Fixed effects of sex and paradigm were compared to a reference (male and 30 s heat/60 s cold, respectively).

Model	Predictor	Estimate	Std. Error	*p*-Value	Multiple R^2^
Model 1	Intercept	−17.385	9.933	0.082	0.0109
	NRS_cond_	−0.916	1.101	0.407	
	Age	−0.053	0.288	0.854	
	Sex	4.181	4.080	0.307	
	30 s heat/60 s cold	3.643	8.853	0.681	
	Electrical/120 s cold	−1.190	4.988	0.812	
Model 2	Intercept	−27.890	10.237	0.007	0.0194
	Temperature_cond_	0.866	0.594	0.147	
	Age	−0.102	0.288	0.723	
	Sex	4.818	4.096	0.242	
	Heat30 s/Cold60 s	1.825	8.715	0.834	
	Electrical/120s cold	0.470	5.141	0.927	

**Table 3:** Relation between CPM effect and CS pain intensity or physical intensity (temperature) in the repeated measures analysis. Linear mixed effect analysis of Model 3 (n = 85 participants, 184 observations) and Model 4 (n = 52 participants, 118 observations), see Methods for model specification. *p*-values were obtained by Wald’s Chi-Square test on the fitted mixed models. NRScond, CS pain intensity rating on the NRS [0–10]. Temperaturecond, temperature of the CS (cold water bath). Paradigm, 30 s heat/60 s cold as opposed to Electrical/cold120s (reference). Repeat refers to the measurement repeat and is used to control for order of measurement. Significant effects are marked in bold.

Model	Predictor	Estimate	Std. Error	*p*-Value	REML Criterium at Convergence
Model 3	Intercept	−11.685	13.461	-	1649.6
	NRS_cond_	−1.485	1.056	0.159	
	Age	0.257	0.388	0.506	
	Sex	2.452	4.402	0.578	
	Paradigm	−7.815	4.808	0.104	
	Repeat	−2.617	2.637	0.321	
Model 4	Intercept	−21.712	13.935	-	1015.8
	**Temperature_cond_**	**1.532**	**0.482**	**0.002**	
	Age	0.258	0.428	0.546	
	Sex	−1.860	4.831	0.700	
	Paradigm	−8.468	4.910	0.085	
	Repeat	−3.073	2.343	0.190	

**Table 4:** Variance in the CPM effect explained by fixed effects, including CS pain and physical intensity in the repeated measures analysis. Model 5: n = 85 participants, 184 observations; model 6: n = 52 participants, 118 observations, see Methods for model specifications. R^2^ indicates the total variance explained by each individual predictor as calculated by the *calc.relimp()* (relaimpo package) in R. NRScond, pain intensity rating of conditioning stimulus on the NRS [0–10]. Temperaturecond, temperature of the conditioning stimulus (cold water bath). Paradigm, 30 s heat/60 s cold as opposed to Electrical/120 s cold (reference). Repeat refers to the measurement repeat and is used to control for order of measurement.

Model 5		Model 6	
Predictor	R^2^	Predictor	R^2^
NRS_cond_	0.00681	Temperature_cond_	0.04650
Age	0.00438	Age	0.00886
Sex	0.00559	Sex	0.00091
Paradigm	0.01491	Paradigm	0.03010
Repeat	0.00325	Repeat	0.00871

## Data Availability

Data is available upon reasonable request from the corresponding author.
